# Development and application of an antibody-based protein microarray to assess physiological stress in grizzly bears (*Ursus arctos*)

**DOI:** 10.1093/conphys/cow001

**Published:** 2016-02-26

**Authors:** Ruth I. Carlson, Marc R. L. Cattet, Bryan L. Sarauer, Scott E. Nielsen, John Boulanger, Gordon B. Stenhouse, David M. Janz

**Affiliations:** 1 Toxicology Graduate Program, University of Saskatchewan, Saskatoon, SK, CanadaS7N 5B3; 2 Canadian Wildlife Health Cooperative, Western College of Veterinary Medicine, University of Saskatchewan, Saskatoon, SK, CanadaS7N 5B4; 3 Toxicology Centre, University of Saskatchewan, Saskatoon, SK, CanadaS7N 5B3; 4 Department of Renewable Resources, 751 General Services, University of Alberta, Edmonton, AL, CanadaT6G 2H1; 5 924 Innes, Integrated Ecological Research, Nelson, BC, CanadaV1L 5T2; 6 Foothills Research Institute and Alberta Sustainable Resource Development, Hinton, AL, CanadaT7V 1V3; 7 Department of Veterinary Biomedical Sciences, University of Saskatchewan, Saskatoon, SK, CanadaS7N 5B4

**Keywords:** Grizzly bear, proteomics, skin biopsy, stress

## Abstract

A novel antibody-based protein microarray was developed that simultaneously determines expression of 31 stress-associated proteins in skin samples collected from free-ranging grizzly bears (*Ursus arctos*) in Alberta, Canada. The microarray determines proteins belonging to four broad functional categories associated with stress physiology: hypothalamic–pituitary–adrenal axis proteins, apoptosis/cell cycle proteins, cellular stress/proteotoxicity proteins and oxidative stress/inflammation proteins. Small skin samples (50–100 mg) were collected from captured bears using biopsy punches. Proteins were isolated and labelled with fluorescent dyes, with labelled protein homogenates loaded onto microarrays to hybridize with antibodies. Relative protein expression was determined by comparison with a pooled standard skin sample. The assay was sensitive, requiring 80 µg of protein per sample to be run in triplicate on the microarray. Intra-array and inter-array coefficients of variation for individual proteins were generally <10 and <15%, respectively. With one exception, there were no significant differences in protein expression among skin samples collected from the neck, forelimb, hindlimb and ear in a subsample of *n* = 4 bears. This suggests that remotely delivered biopsy darts could be used in future sampling. Using generalized linear mixed models, certain proteins within each functional category demonstrated altered expression with respect to differences in year, season, geographical sampling location within Alberta and bear biological parameters, suggesting that these general variables may influence expression of specific proteins in the microarray. Our goal is to apply the protein microarray as a conservation physiology tool that can detect, evaluate and monitor physiological stress in grizzly bears and other species at risk over time in response to environmental change.

## Abstract (precis)

A novel antibody-based protein microarray was developed that determines expression profiles of 31 stress-associated proteins in small skin biopsies collected from free-ranging grizzly bears. Laboratory and preliminary field validation of the microarray suggests that it may provide a useful tool for conservation physiology research involving bears and other wildlife species.

## Introduction

Anthropogenic landscape modification is considered to be the principal threat to global biodiversity (Lindenmayer and Fischer, 2006). Climate change, habitat loss and habitat fragmentation impact many organisms, but species that are highly specialized, sensitive to disturbance and/or dependent upon particular ecological conditions at specific times of the year appear most vulnerable (Wittmer *et al*., 2007; Durner *et al*., 2009). Limited reproductive capacity, low population density and large home range requirements make large mammalian carnivores, such as grizzly bears (*Ursus arctos*), inherently more susceptible to environmental change (Weaver *et al*., 1996). Grizzly bear populations in Alberta, Canada have been studied extensively over the past 15 years and have been listed by the provincial government as threatened (Alberta Sustainable Resource Development, 2010). It has been predicted that grizzly bears will continue to experience habitat alterations and population declines in Alberta (Benn and Herrero, 2002; Nielsen *et al*., 2004b, 2008; Boulanger and Stenhouse, 2014), thus threatening the sustainability of certain populations.

Landscape change may not only have a direct effect on wildlife by altering habitats, but may also have an indirect effect more difficult to measure by acting as a source of long-term physiological stress. In disturbed areas, normal behaviour, life-history traits and intra- or inter-specific interactions may be altered, forcing wildlife species to cope with stressors exceeding those encountered in unaltered habitat (Wikelski and Cooke, 2006). Although the physiological response to short-term stress is adaptive, long-term stress (lasting weeks to months) may lead to a pathological syndrome of impaired reproduction, immunosuppression and diminished growth (Moberg, 2000; Boonstra, 2005; Wikelski and Cooke, 2006). Measures of population performance (i.e. demographic rates) and abundance may be affected negatively as the proportion of individuals in a population experiencing impaired health owing to long-term stress increases (Sheriff *et al*. 2011; Perez-Heydrich *et al*. 2012). However, most evidence for linkages between long-term stress and pathology arises from biomedical literature. It has recently been argued that in certain wild species, long-term stress arising from natural stressors may be adaptive and continue to promote fitness (Boonstra, 2013). Long-term physiological stress in individual animals may therefore be an important mechanism linking environmental change with impaired wildlife health. There is, however, a need for better understanding of both positive and negative influences of long-term stress in free-ranging wildlife.

If wildlife is adversely affected by anthropogenic landscape change, biological markers of long-term physiological stress should be measurable in individuals before adverse effects are apparent at the population level. Reliable tools to assess long-term stress in free-ranging wildlife are lacking, and the development of such techniques will enhance our understanding of the effects of environmental stressors on wildlife (Reeder and Kramer, 2005; Russell *et al*., 2012). The development and validation of such tools would facilitate the monitoring of population performance and might provide an opportunity to alleviate environmental stressors before they impact populations. The term ‘ecological forecasting’ has been used to describe the need for novel markers of physiological function that can be used for the conservation and recovery of wildlife species at risk (Clark *et al*., 2001).

Protein microarrays are a promising tool for analysis of multiple protein-level changes in cells responding to varying stimuli and have been successfully used for quantitative proteomics, clinical diagnostics and biomarker-directed drug discovery in human biomedical sciences (Cutler, 2003; Haab, 2003; Kingsmore, 2006; Sun *et al*., 2013). As a logical evolution of technology used in miniaturized DNA assemblies on chips, protein microarrays consist of a series of capture molecules (usually antibodies) that are spotted onto known locations on a matrix, allowing the determination of increased or decreased protein expression compared with a reference sample. The need for protein arrays stems from the often poor correlation observed between mRNA and protein expression in cells (Cutler, 2003). Advantages of protein microarrays over traditional protein separation and identification techniques include enhanced throughput, speed and sensitivity and minimal sample consumption. Minimal sample consumption is especially beneficial for monitoring wildlife in a non-lethal manner.

The objective of the present study was to develop and validate an antibody-based protein microarray that can detect changes in a suite of stress-associated proteins in skin biopsy samples collected from free-ranging grizzly bears in Alberta. The broader goal was to develop a monitoring tool to assist wildlife managers in identifying and evaluating grizzly bear populations at risk, with potential application to other wildlife species.

## Materials and methods

### Animal capture, tissue collection ’and sample processing

The Foothills Research Institute (FRI) Grizzly Bear project captured grizzly bears in western Alberta, Canada encompassing the area from the Montana, USA border north into the boreal forest and centrally to the Swan Hills from 2004 to 2012. Capture locations represented six distinct bear management areas (BMAs) within Alberta (Swan Hills, Grande Cache, Yellowhead, Clearwater, Livingstone and Castle), which are delineated by major east–west highways (Cattet *et al*., 2014). Capture methods included leg-hold snare, remote drug delivery from helicopter, and culvert traps (see Cattet *et al*., 2008 for a detailed capture and handling protocol). A premolar tooth was extracted from bears captured for the first time and appearing to be older than 1 year, to estimate age by counting cementum annuli (Stoneberg and Jonkel, 1966). All skin samples (*n* = 139) were collected from captured bears using a biopsy punch (Miltex Inc., York, PA, USA) or biopsy dart (Paxarms NZ Ltd, Timaru, New Zealand). Samples were immediately placed in a cooler on ice, frozen within 1–4 h in liquid nitrogen or on dry ice, and kept frozen until storage at −80°C at the University of Saskatchewan. This protocol was approved by the University of Saskatchewan University Committee on Animal Care and Supply and by the Animal Care Committee of Alberta Environment and Sustainable Resource Development each year for the duration of the project. The protocol was in accordance with guidelines of the American Society of Mammalogists Animal Care and Use Committee (Sikes *et al*., 2011) and the Canadian Council on Animal Care (CCAC, 2003).

In addition to the small (approximately 50–100 mg) skin biopsy samples collected from captured bears, larger pieces of skin (approximately 50–100 g) were opportunistically collected from six bears that died through bear management measures or self-defense actions or were killed by another predator. The larger bulk quantities of skin from these bears were used for development of the stress protein microarray. Before storage at −80°C, any external hair and attached muscle tissue on all skin samples was removed using a scalpel. Protein isolation and labelling and the microarray procedure followed methods outlined by Haab and Zhou (2004). Frozen grizzly bear skin samples were ground to powder under liquid nitrogen using a pre-chilled mortar and pestle. Proteins were isolated from ground samples by adding 10 ml of lysis buffer [50 mM HEPES pH 7.0, 5 mM EDTA, 50 mM NaCl, 10 mM sodium pyrophosphate, 50 mM NaF, 10 mM sodium vanadate, 1% Nonidet P-40 and complete protease inhibitor (Roche, Toronto, ON, Canada)] per gram of tissue and incubating for 15 min on ice. After centrifuging the lysed samples “at 5000 *g* for 20 minutes at 4C,” the supernatant was collected, concentrated using centrifugal filters (Ultracel YM-10; Millipore, Bedford, MA, USA) and stored at −80°C. Protein concentrations were determined using a modified Lowry *et al*. (1951) assay (DC protein assay; BioRad, Hercules, CA, USA).

### Identification of antibodies for use in the microarray

To identify a suite of commercially available antibodies that specifically recognized stress-associated proteins in grizzly bear skin, 285 antibodies from 19 companies were screened using western blotting. Isolated proteins from the bulk skin samples were denatured and separated by size with sodium dodecyl sulfate–polyacrylamide gel electrophoresis using 7.5, 12.5 or 15% acrylamide gels. Proteins in each gel were transferred to 0.22 µm nitrocellulose membranes (GE Healthcare, Picataway, NJ, USA) and detected using enhanced chemiluminescence (ECL Plus; GE Healthcare). The size of the resultant band(s) was determined through comparison with molecular standards (Kaleidoscope prestained standards 161-0324 or Precision Plus Kaleidoscope 161-0375; BioRad), and only those antibodies that recognized protein bands of the correct molecular weight were selected as positive. Additionally, those antibodies that recognized additional non-specific bands were rejected. This resulted in the identification of 31 antibodies for subsequent microarray development based on those displaying a strong signal and low non-specific binding (Table 1). The stress-associated proteins recognized by these antibodies were classified into four functional categories: hypothalamic–pituitary–adrenal (HPA) axis proteins, apoptosis and cell cycle (ACC) proteins, cellular stress and proteotoxicity (CSP) proteins and oxidative stress and inflammation (OSI) proteins (Table 1).

**Table 1:** List of 31 stress-associated proteins, separated by functional category, detected using the microarray

Category	Proteins
Hypothalamic–pituitary–adrenal (HPA) axis	Adrenocorticotropic hormone (ACTH; Biodesign, BDE54057 M), arginine vasopressin receptor 1a (AVPR1A; Santa Cruz, sc30025), corticotrophin-releasing hormone receptor 1/2 (CRHR-1/2; Santa Cruz, sc5543), glucocorticoid receptor (GR; Santa Cruz, sc1002), C-terminal proopiomelanocortin (POMC) precursor (Abcam, ab32893), prolactin (Santa Cruz, sc7805)
Apoptosis and cell cycle (ACC)	Apoptosis inducing factor (AIF; Santa Cruz, sc13116), annexin II (Santa Cruz, sc1924), annexin IV (Santa Cruz, sc1930), caspase 1 (Santa Cruz, sc514), caspase 2 (Labvision, rb1699), caspase 6 (Sigma, c7599), epithelial (E)-cadherin (Santa Cruz, sc31020), glyceraldehyde-3-phosphate dehydrogenase (GAPDH; Assay Designs, 905-734-100)
Cellular stress and proteotoxicity (CSP)	Cytokeratin (Abcam, ab9377), glucose regulated protein 78 (GRP78/BiP; Sigma, G9043), heat shock protein (HSP)27 (Stressgen, SPA524), HSP40 (Sigma, H4038), HSP60 (Sigma, H3524), HSP70 (Santa Cruz, sc24), HSP70 inducible (HSP70i; Stressgen, SPA810), HSP90 (Stressgen, SPS771) HSP110 (Sigma, H7412)
Oxidative stress and inflammation (OSI)	C-C chemokine receptor 5 (CCR5; Sigma, C8604), cyclooxygenase-2 (COX-2; Santa Cruz, sc7951), haem oxygenase-2 (HO-2; Santa Cruz, sc11361), endothelial nitric oxide synthase (eNOS; Abcam, ab5589), inducible nitric oxide synthase (iNOS; Sigma, N7782), peroxiredoxin-3 (PRDX3; Sigma, P1247), superoxide dismutase (SOD) 1 (Santa Cruz, sc8637), SOD2 (Abcam, ab13533)

Abbreviations, commercial antibody suppliers and catalogue numbers are in parentheses.

### Protein microarray development

Microarrays were printed (i.e. antibodies immobilized onto 3 cm × 8 cm glass slides) by First Phase Technologies (Tempe, AZ, USA) onto Full Moon BioSystems (Sunnyvale, CA, USA) protein array substrate slides. Initially, a prototype microarray was printed and used to test methods of isolating replicate arrays on each slide, selecting blocking buffers and wash buffers, selecting the appropriate antibody dilution and optimizing the time of incubation. Once this initial validation was completed, final protein microarrays were produced. Six replicate microarrays were printed onto each slide, with each array consisting of 36 spots in a 6 × 6 grid consisting of the 31 antibodies specific for grizzly bear stress proteins. The remaining five spots on each array consisted of a negative control (print buffer), a positive control (Cy5-labelled protein supplied by the manufacturer) and a dilution series of anti-cytokeratin antibody printed at 1:1, 1:5 and 1:25 dilutions in print buffer. Based on results from the antibody dilutions on the prototype microarrays, all other antibodies were diluted 1:1 in print buffer. Once printed, microarrays were stored at room temperature in a sealed desiccator until use.

In order to detect proteins that were specifically bound to the antibodies on the array, proteins were labelled with the fluorescent cyanine dyes Cy3 and Cy5 following the protocol provided by the manufacturer (GE Healthcare). Portions of the six bulk samples of bear skin were homogenized separately, and an equal amount of protein from each of the six bears was combined to create a pooled standard, which was labelled with Cy3. Individual bear samples were labelled with Cy5, and the relative fluorescence compared with the Cy3-labelled pooled standard was determined.

### Microarray procedure

Silicone isolators (Grace Bio-Labs, Bend, OR, USA) were clamped onto microarray slides in order to separate the six replicate arrays and create discrete wells on the slide. Arrays were blocked prior to use by incubation with 1% bovine serum albumin, rinsed with double-distilled water (ddH_2_O), then washed five times with phosphate-buffered saline containing 0.5% Tween-20 (0.5% PBST, pH 7.4), washed three more times with ddH_2_O and finally dried under a gentle stream of N_2_. Equal amounts of protein (80 µg) from the dye-labelled pooled standard and individual bear sample were combined and then added to each of three arrays on a slide, with two individual bear samples applied per slide. Thus, each sample was run in triplicate if sufficient sample protein was available, which occurred with 136 of 139 skin samples. The remaining three samples were run in duplicate. The hybridization reaction was incubated for 1 h with agitation, then rinsed sequentially with 0.1% PBST and ddH_2_O and dried under N_2_ before scanning.

### Microarray scanning

Array scanning was conducted using an Axon Instruments GenePix 4000B scanner (Molecular Devices, Sunnyvale, CA, USA) and GenePix Pro 6.1 software. Scans were performed at 635 and 532 nm, the excitation wavelengths of Cy5 and Cy3, respectively. Scanned images of each slide were carefully checked for saturated pixels, missing or malformed spots, scratches, debris and background inconsistencies that might affect the spot values (Kingsmore, 2006; Romanov *et al*., 2014). Scanned fluorescence values of each of 31 stress-associated proteins from each individual grizzly bear sample run in triplicate (for 136 bears) or duplicate (for three bears) on the microarray were standardized by dividing by the fluorescence value obtained from the pooled grizzly bear standard. Thus, each grizzly bear sample produced triplicate or duplicate values for the expression of each stress-associated protein in relationship to the same standard sample. These triplicate or duplicate values were averaged to provide a single relative protein expression value to be used for statistical analyses.

### Laboratory validation

A series of preliminary laboratory validation experiments were conducted using bulk skin samples before running samples from individual bears biopsied in the field. As measures of precision, a bulk skin sample was initially run on two separate microarrays, and the coefficient of variation (SD/mean × ’100%) was calculated to determine intra-array (*n* = 6) and inter-array (*n* = 12) variation for each of the 31 proteins.

#### Antibody dilution and protein quantity experiments

A dilution series of an anti-cytokeratin antibody was printed on each array at dilutions of 1:1, 1:5 and 1:25 in print buffer and used to determine the potential effect of antibody dilution on measured relative cytokeratin expression. In addition, various quantities of protein (80, 20 and 10 µg) obtained from *n* = 4 bulk skin samples were run on microarrays in order to determine the relative sensitivity when using small quantities of protein that might be obtained from samples collected in the field.

#### Protein degradation and tissue preservative experiments

A potential application of the protein microarray in the conservation biology of grizzly bears is the ability to use incidental samples that become available periodically, such as management kills, self-defense kills and recent road or train kills. In these situations, immediate refrigeration or freezing of tissue samples is often not possible. Thus, to determine what effect time at ambient temperature would have on the protein expression in tissues, bulk skin samples from *n* = 3 bears were subsectioned and held at room temperature for 0, 4, 8, 12, 24 or 48 h. At the end of each time at room temperature, samples were flash frozen in liquid nitrogen and then processed as described previously.

RNA*later*^®^ (Applied Biosystems, Foster City, CA, USA) is a preservative that retards RNA degradation and is commonly used by researchers involved in wildlife studies. To determine the potential effects of RNA*later*^®^ on protein expression and determine whether it could slow protein degradation at ambient temperature, skin samples from *n* = 3 bears were subsectioned, either immersed in 400 µl of RNA*later*^®^ (preserved) or placed in a capped vial (unpreserved), held at room temperature for 24 h and then flash frozen in liquid nitrogen. Protein expression was determined in preserved and unpreserved samples held for 24 h in comparison to subsectioned samples thawed on ice and not subjected to time at room temperature.

#### Comparison of skin sampling locations

To assess whether body location had an effect on protein expression in skin biopsies, multiple locations on the body [ear, neck, forelimb (in the area of the triceps muscle) and hindlimb (in the area of the quadriceps muscles)] were sampled from a subset of *n* = 4 bears and compared using the microarray.

### Statistical analyses of laboratory ’validation experiments

We used generalized linear mixed models (GLMMs; Zuur *et al*., 2013) to evaluate the effects of antibody dilution, protein quantity, time stored at ambient temperature, tissue preservative and skin sampling location. The relationships between response (mean protein expression) and independent variables were modelled using a γ distribution and log link because protein expression values are always positive and usually distributed with a positive skew. For expediency, we modelled relationships on the basis of the four functional groups rather than the 31 individual proteins, except for the antibody dilution experiment, which focused on the expression of a single protein (cytokeratin). Although this assumed that each protein within a functional group would respond in the same manner, we recognized that this might not always be the case. Protein was therefore also included in models as a fixed factor in addition to the potential effects of interest (i.e. antibody dilution, protein quantity, etc). The source of skin samples and individual identity of bears were included in the models as random effects. We used the ‘glmer’ function in package ‘lme4’ (Bates *et al*., 2014) in R 3.1.2 (R Core Team, 2014) for model development. For models in which the potential effect of interest was significant (*P* ≤ 0.05), we compared mean protein expression among all possible pairs by Tukey’s HSD (honest significant difference) test using the ‘glht’ function in package ‘multcomp’ (Hothorn *et al*., 2008) in R 3.1.2 (R Core Team, 2014).

### Associations between protein expression, biology, location and season

As an initial step towards our goal of validating the protein microarray as a conservation tool, we used GLMMs (Zuur *et al*., 2013) to evaluate the effects of biology (sex and age), location (BMA), year (2004–2012, excluding 2009) and season (hypophagia, early hyperphagia and late hyperphagia) on the mean expression for each of 31 proteins. Sex was divided into three categories as male, solitary female and adult female accompanied by dependent offspring. Geographical location, year and season were considered as broad surrogate measures representing numerous environmental factors that we will evaluate in greater depth in future analyses. We used seasons based on grizzly bear feeding habits as defined by Nielsen *et al*. (2004a), where: (i) hypophagia is the period from den emergence (typically in April) to 14 June; (ii) early hyperphagia is from 15 June to 7 August; and (iii) late hyperphagia is from 8 August to den entry, which is typically in November. The unique identity of 119 bears sampled at 139 captures (i.e. multiple samples from some bears) was included in the models as a random effect, as was the batch identity of protein microarrays to adjust for potential differences between batches. For these analyses, we selected the most parsimonious model of eight possible *a priori* combinations of variables (Table 2), including a null model, based on differences in the Akaike’s information criteria corrected for small sample sizes (ΔAIC_c_; Anderson, 2008). We used the ‘glmer’ function in package ‘lme4’ (Bates *et al*., 2014) for model development in R 3.1.2 (R Core Team, 2014). Our intent with these analyses was simply to determine whether skin protein expression in grizzly bears was influenced by biological and/or environmental factors.

**Table 2:** Models selected *a priori* to evaluate the effects of biology (sex and age), location (bear management area), year (2004–2012, excluding 2009) and season (hypophagia, early hyperphagia and late hyperphagia) on the mean expression for each of 31 proteins

Number	Description	Fixed effects	Random effects
1	Null (intercept only)		Bear, batch
2	Biology	Sex + age + (sex × age)	Bear, batch
3	Location	BMA	Bear, batch
4	Time	Year + season	Bear, batch
5	Biology and location	Sex + age + (sex × age) + BMA	Bear, batch
6	Biology and time	Sex + age + (sex × age) + season + (sex × season)	Bear, batch
7	Location and time	BMA + year	Bear, batch
8	Global	Sex + age + (sex × age) + BMA + year + season + (sex × season)	Bear, batch

Sex includes ‘adult female accompanied by dependent offspring’ as a third category. Abbreviation: BMA, bear management area.

## Results

### Laboratory validation

A total of 285 commercially available antibodies to stress-associated proteins were evaluated for their ability to cross-react with proteins in grizzly bear skin. Of these, 31 antibodies recognized grizzly bear proteins and were used to develop the protein microarray (Table 1). Polyclonal antibodies made up the majority (26 of 31) of antibodies selected for the microarray. Based on their primary functions, each protein was classified into one of the following four categories: (1) proteins associated with the hypothalamic–pituitary–adrenal (HPA) axis; (2) proteins associated with apoptosis and cell cycle (ACC); (3) proteins associated with cellular stress and proteotoxicity (CSP); and (4) proteins associated with oxidative stress and inflammation (OSI; Table 1).

A series of laboratory validation experiments were conducted to determine the performance of the microarray. To determine the consistency of protein expression obtained within a microarray slide (consisting of six individual arrays) and between microarray slides (12 individual arrays), intra-array and inter-array variation, respectively, was determined. Intra-array variation was <10% for 28 of 31 proteins, and between 10–15% and for three of 31 proteins (data not shown). Inter-array variation was <15% for 27 of 31 proteins, and between 15 and 18% for four of 31 proteins (data not shown).

**Figure 1:**
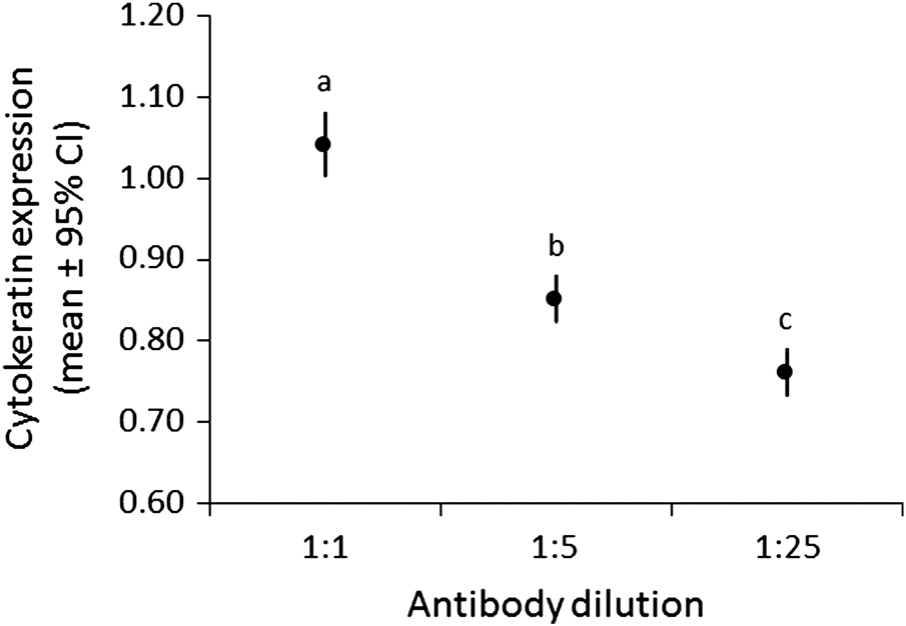
Mean relative cytokeratin expression in 82 grizzly bear skin samples in relationship to three different dilutions (1:1, 1:5 and 1:25) of an anti-cytokeratin antibody. Significant differences (*P* ≤ 0.05) between means are based on Tukey’s HSD test and are indicated by a > b > c.

The anti-cytokeratin antibody was printed on each microarray at 1:1, 1:5 and 1:25 dilutions in printing buffer. Increasing dilution of anti-cytokeratin antibody had a significant effect on measured cytokeratin expression (GLMM, *P* ≤ 0.001, *n* = 82 skin samples; Fig. 1). Each antibody dilution was significantly different from each other, and there was decreased cytokeratin expression with increasing dilution (Tukey’s HSD test, *P* ≤ 0.001). In addition, inconsistencies in spot morphology (reduced size, irregular shape and missing centre) were commonly observed with increasing antibody dilutions.

**Figure 2:**
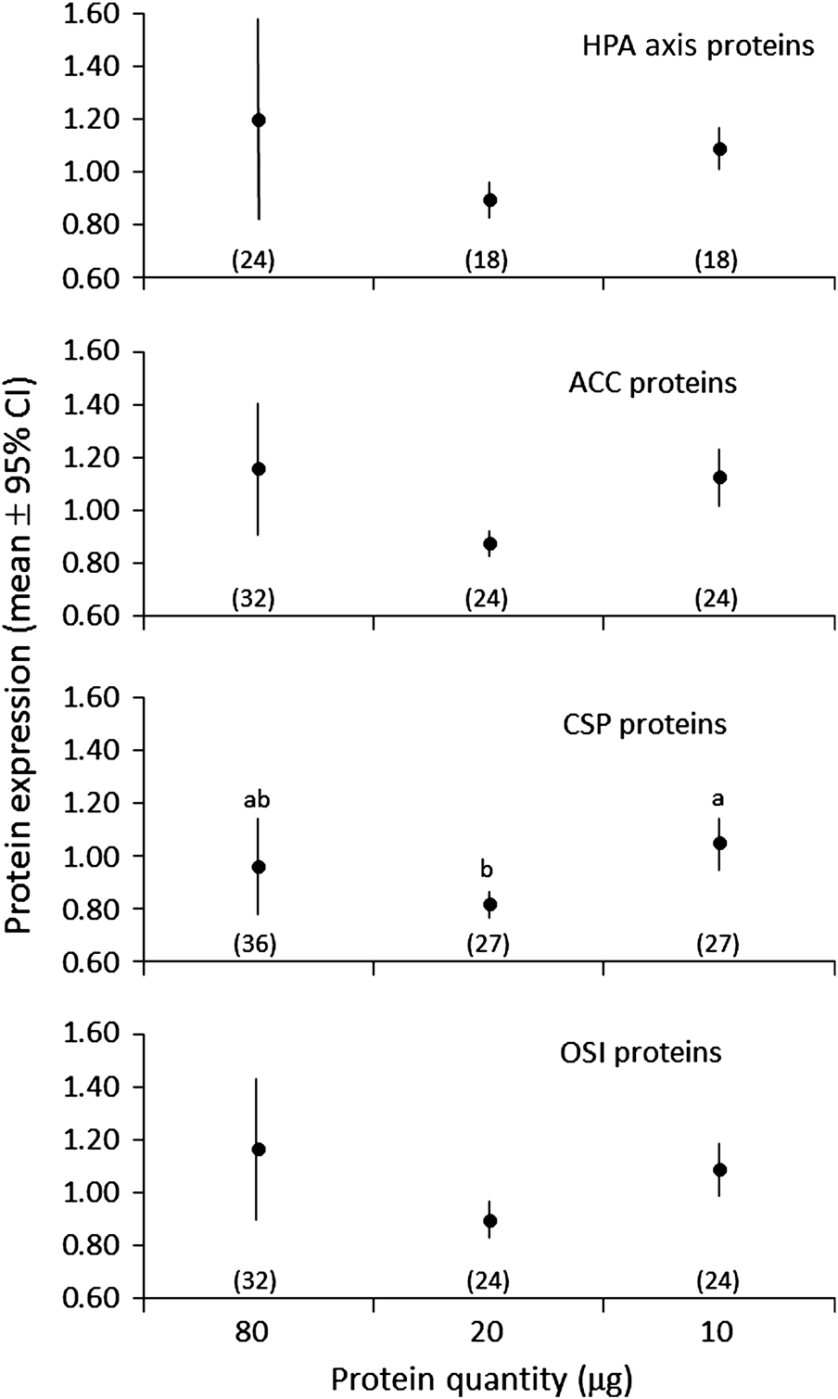
Mean relative protein expression in relationship to different quantities of protein isolated from skin samples collected from four grizzly bears. The number of observations at each protein quantity is provided in parentheses and was calculated as the number of skin samples (four) multiplied by the number of proteins per functional group (HPA axis, 6; ACC, 8; CSP, 9; and OSI, 8) minus the number of missing values. Significant differences (*P* ≤ 0.05) between means are based on Tukey’s HSD test and are indicated by a > b, a ≥ ab and ab ≥ b. Abbreviations: ACC, apoptosis and cell cycle; CSP, cellular stress and proteotoxicity; HPA, hypothalamic–pituitary–adrenal; and OSI, oxidative stress and inflammation.

Processing of the 50–100 mg skin biopsy samples from individual bears captured in the field consistently provided yields of 80 µg of protein, which allowed each sample to be loaded in triplicate on each microarray. To determine whether lesser quantities of protein would provide similar protein expression levels, 10, 20 or 80 µg of protein were run on the microarray. For HPA axis and OSI protein categories, mean protein expression was similar among protein quantities (Tukey’s HSD test, *P* > 0.12 for three categories; Fig. 2). Mean protein expression was also similar among protein quantities in the ACC protein category, but expression with the 20 µg quantity was only marginally non-significant in comparison with expression with the 10 (Tukey’s HSD test, *P* = 0.09) and 80 µg quantities (Tukey’s HSD test, *P* = 0.08). For CSP proteins, less protein expression was observed with 20 rather than 10 µg of loaded protein (Tukey’s HSD test, *P* = 0.04).

Two internal control spots were included within each microarray in an attempt to allow standardization among arrays. The negative control spot consisted of a single print buffer, but inconsistencies in spot morphology, size and other irregularities during scanning did not allow it to be used consistently as an internal control for potential background fluorescence. The positive control consisted of a Cy5-labelled protein that was supplied by the manufacturer during microarray printing. However, this spot did not fluoresce during scans of microarrays at the appropriate excitation wavelength, possibly because of dye degradation. For these reasons, the internal controls were not used to standardize among microarrays when analysing individual grizzly bear skin samples.

**Figure 3:**
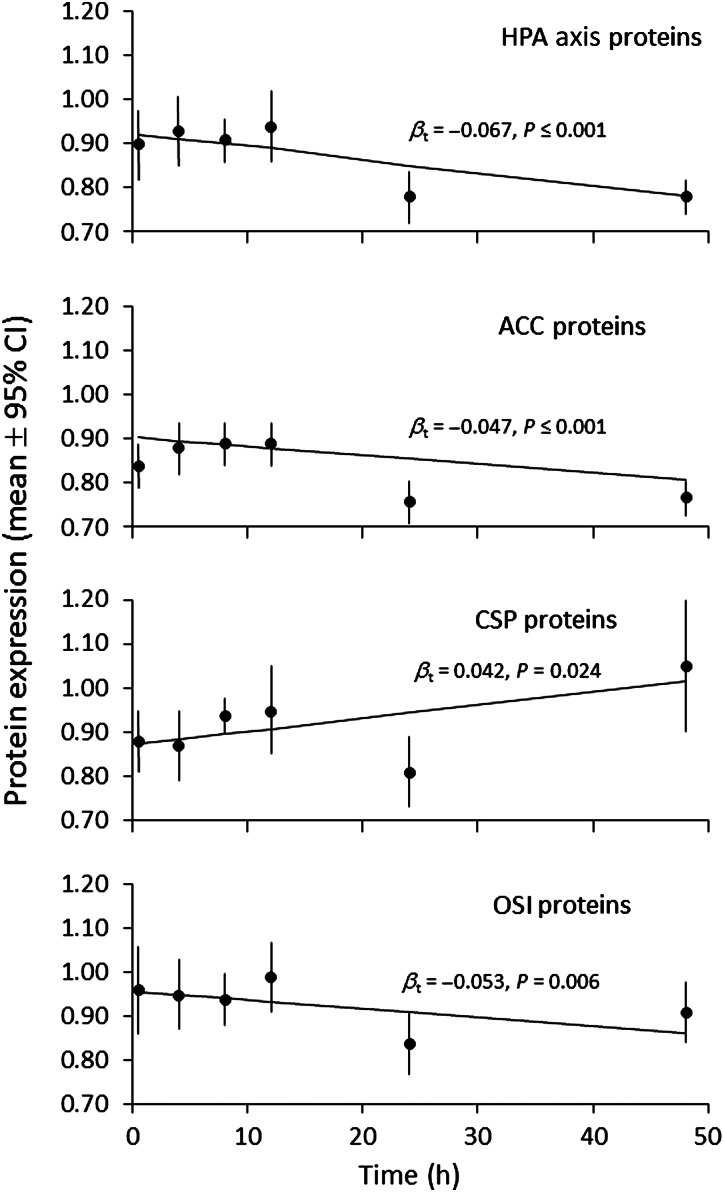
The effect of storage at 21°C for different periods of time (0, 4, 8, 12, 24 or 48 h) prior to flash freezing on the mean relative protein expression in skin samples collected from three grizzly bears. The number of observations at each time point ranged from 18 to 27 and was calculated as the number of skin samples (three) multiplied by the number of proteins per functional group (HPA axis, 6; ACC, 8; CSP, 9; and OSI, 8). There were no missing values. The time coefficients (β*_t_*) and their statistical significance were determined by including time (*t*) as a covariate in generalized linear mixed models, one for each functional group, which also included the group-specific proteins as a fixed effect and the individual bears as a random effect. However, the lines were determined by simple linear regression between protein expression and time and are intended only as a visual aid. Abbreviations are as in the legend to Fig. 2.

Although immediate freezing of skin samples collected from grizzly bears in field studies is ideal, in practice this is not always logistically possible. Thus, it was of interest to determine the protein expression levels for subsections of skin from three grizzly bears that varied in time (4–48 h) held at room temperature before frozen storage. For HPA axis, ACC and OSI proteins, an inverse significant relationship with time at room temperature was found (GLMMs, *P* ≤ 0.001, *P* ≤ 0.001 and *P* = 0.006, respectively; Fig 3). For CSP proteins, time at room temperature was directly related to protein expression (GLMM, *P* = 0.024; Fig. 3).

**Figure 4:**
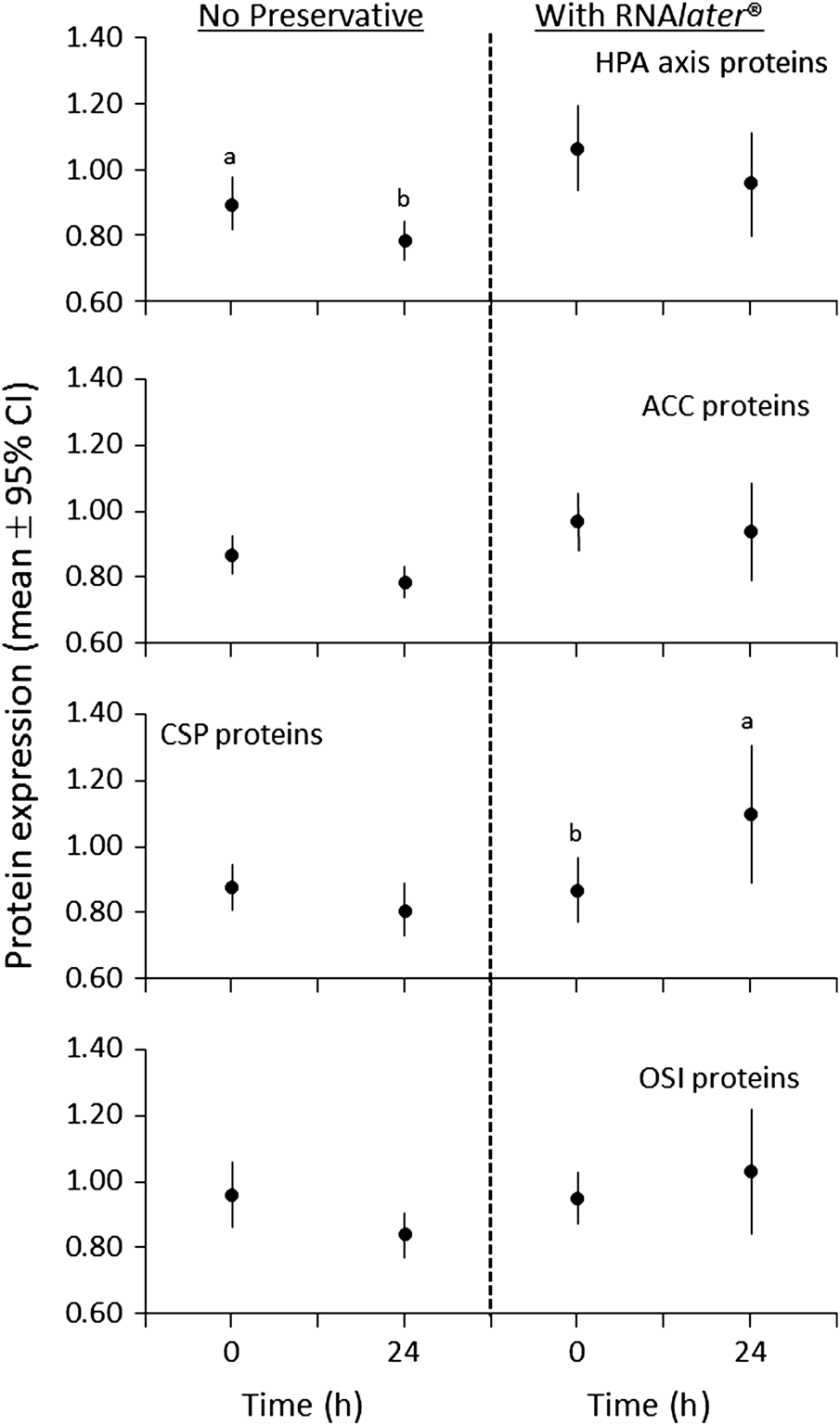
The effect of an RNA stabilization and storage reagent (RNA*later*^©^) on the mean relative protein expression in skin samples that were collected from three grizzly bears and held at 21°C for 24 h before flash freezing. The number of observations at each treatment ranged from 18 to 27 and was calculated as the number of skin samples (three) multiplied by the number of proteins per functional group (HPA axis, 6; ACC, 8; CSP, 9; and OSI, 8) minus the number of missing values. Significant differences (*P* ≤ 0.05) between means are based on Tukey’s HSD test and are indicated by a > b. Abbreviations are as in the legend to Fig. 2.

To determine what effect the commonly used preservative RNA*later*^®^, in comparison to no preservative, might have on protein expression in samples that had undergone potential protein degradation, an experiment was conducted with subsectioned grizzly bear skin from three individual bears subjected to 24 h at room temperature either immersed in RNA*later*^®^ or unpreserved. For unpreserved samples, there was significantly less HPA axis protein expression at 24 than at 0 h (Tukey’s HSD test, *P* = 0.040), with a similar but non-significant trend for ACC (*P* = 0.126), CSP (*P* = 0.547) and OSI proteins (*P* = 0.083; Fig. 4). For CSP proteins treated with RNA*later*^®^, there was significantly greater protein expression at 24 than at 0 h (*P* = 0.032; Fig. 4).

**Figure 5:**
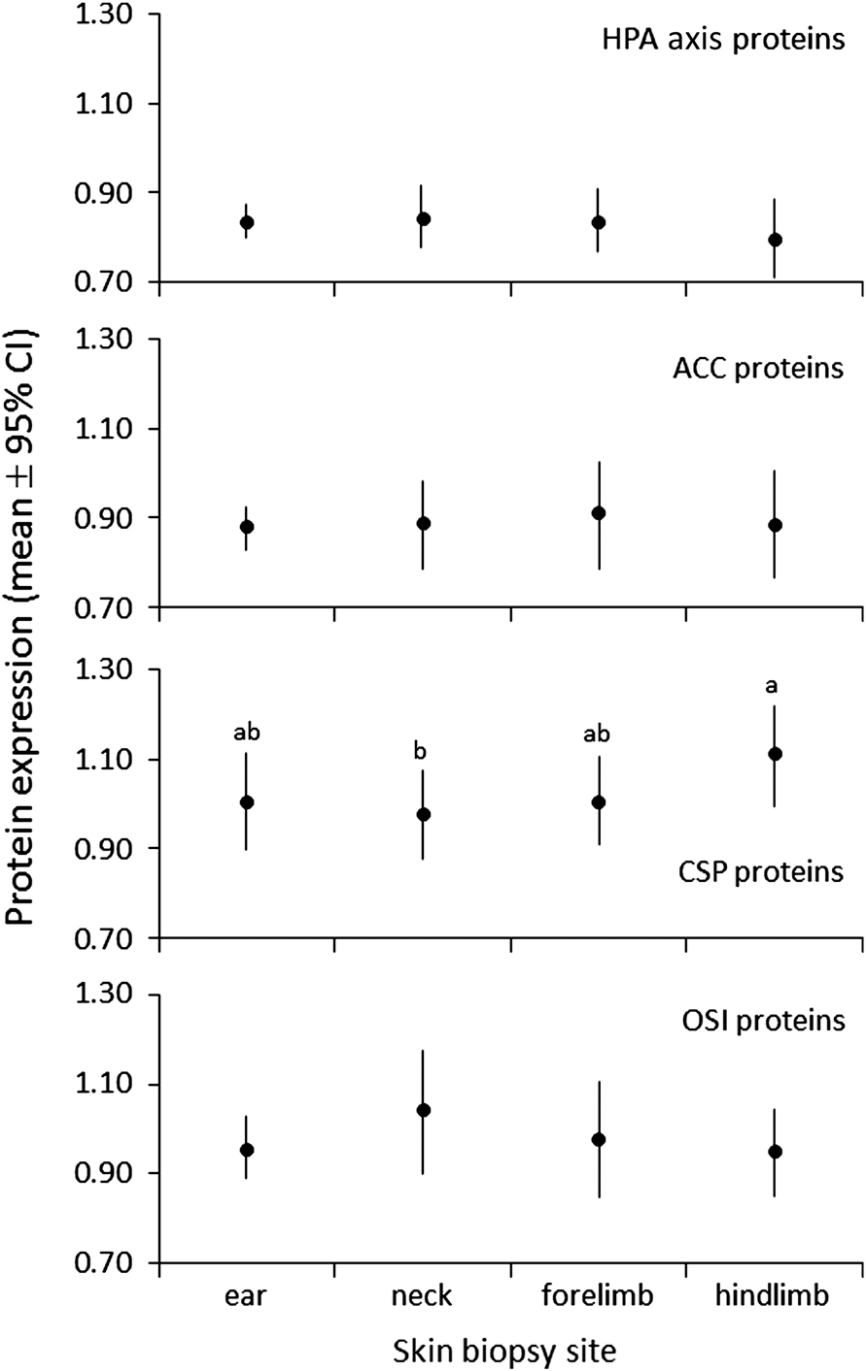
The effect of biopsy location (ear, neck, forelimb or hindlimb) on the mean relative protein expression in skin samples that were collected from four grizzly bears. The number of observations at each protein quantity ranged from 24 to 36 and was calculated as the number of skin samples (four) multiplied by the number of proteins per functional group (HPA axis, 6; ACC, 8; CSP, 9; and OSI, 8). There were no missing values. Significant differences (*P* ≤ 0.05) between means are based on Tukey’s HSD test and are indicated by a > b, a ≥ ab and ab ≥ b. Abbreviations are as in the legend to Fig. 2.

Samples from different skin locations on four grizzly bears (ear, neck, forelimb and hindlimb) were evaluated to determine whether there were differences in protein expression according to body location. There were no significant differences in protein expression among skin locations for HPA axis, ACC or OSI proteins (Tukey’s HSD tests, *P* ≥ 0.595; Fig. 5). Protein expression of CSP proteins was significantly greater in skin collected from hindlimb compared with neck (*P* = 0.015; Fig. 5).

Although protein expression in association with protein quantity, time stored at ambient temperature, tissue preservative and skin sampling location was generally similar among individual proteins within functional categories (results not shown), protein expression differed based mostly on location and year, but also to a lesser degree on bear biology and season (Tables 3–6). Either a time-only model (model 4 in Table 2) or a location and time model (model 7 in Table 2) was selected as the top model for all but two of 31 proteins (Tables 3–6), with time consistently selected as an influential variable in all top models.

**Table 3:** Standardized coefficients and credible intervals of model variables describing hypothalamic–pituitary–adrenal axis protein expression in skin biopsy samples collected from 119 grizzly bears over 139 captures in western Alberta, Canada, from 2004 to 2012

Predictor variables	Hypothalamic–pituitary–adrenal axis stress proteins (standardized coefficient and credible interval)					
	ACTH	AVPR1A	CRFR-1/2	GR	POMC	Prolactin
(1) Intercept	***−0.339 (−0.661 to −0.017)***	−0.424 (−0.922 to 0.075)	***−0.469 (−0.837 to −0.102)***	−0.438 (−0.880 to 0.004)	***−0.956 (−1.393 to −0.519)***	***−0.420 (−0.823 to −0.017)***
(2) Year						
2012	0.227 (−0.195 to 0.648)	0.362 (−0.321 to 1.046)	0.421 (−0.064 to 0.906)	0.474 (−0.120 to 1.068)	0.196 (−0.077 to 0.470)	0.450 (−0.091 to 0.990)
2011	0.024 (−0.414 to 0.463)	0.346 (−0.346 to 1.038)	0.363 (−0.134 to 0.861)	0.473 (−0.133 to 1.078)	0.064 (−0.246 to 0.373)	0.419 (−0.133 to 0.971)
2010	0.068 (−0.557 to 0.694)	0.468 (−0.341 to 1.276)	0.287 (−0.311 to 0.886)	0.537 (−0.196 to 1.270)	−0.251 (−0.918 to 0.417)	0.544 (−0.143 to 1.230)
2008	0.045 (−0.113 to 0.203)	−0.018 (−0.176 to 0.140)	0.020 (−0.136 to –0.176)	−0.022 (−0.194 to 0.150)	0.164 (−0.056 to 0.383)	−0.031 (−0.192 to 0.130)
2007	0.034 (−0.140 to 0.207)	0.145 (−0.044 to 0.333)	0.132 (−0.052 to 0.316)	0.087 (−0.118 to 0.291)	0.145 (−0.114 to 0.405)	0.0645 (−0.128 to 0.257)
2006	***0.176 (0.007–0.344)***	0.182 (−0.0002 to 0.364)	***0.230 (0.051–0.409)***	0.192 (−0.006 to 0.390)	0.237 (−0.018 to 0.492)	0.150 (−0.035 to 0.336)
2005	***0.258 (0.101–0.415)***	***0.250 (0.075–0.425)***	***0.279 (0.108–0.451)***	***0.269 (0.079–0.458)***	***0.416 (0.172–0.660)***	***0.203 (0.024–0.381)***
2004	0	0	0	0	0	0
(3) Bear management area						
Grande Cache		0.042 (−0.133 to 0.216)	0.014 (−0.165 to 0.192)	0.046 (−0.147 to 0.238)	***0.323 (0.067–0.578)***	0.033 (−0.146 to 0.212)
Swan Hills		0.122 (−0.111 to 0.355)	0.067 (−0.175 to 0.309)	0.123 (−0.137 to 0.382)	***0.383 (0.035***–***0.731)***	0.123 (−0.117 to 0.363)
Yellowhead		0.107 (−0.087 to 0.300)	0.089 (−0.112 to 0.290)	0.116 (−0.099 to 0.331)	***0.439 (0.155–0.722)***	0.111 (−0.088 to 0.310)
Clearwater		0.127 (−0.078 to 0.332)	0.132 (−0.081 to 0.345)	0.119 (−0.109 to 0.347)	***0.434 (0.139–0.730)***	0.098 (−0.113 to 0.309)
Livingstone		−0.079 (−0.288 to 0.129)	−0.118 (−0.332 to 0.096)	−0.108 (−0.338 to 0.122)	−0.038 (−0.331 to 0.254)	−0.123 (−0.337 to 0.091)
Castle		0	0	0	0	0
(4) Sex						
Male					0.420 (−0.030 to 0.870)	
Female with young					0.144 (−0.289 to 0.577)	
Solitary female					0	
(5) Age					0.027 (−0.059 to 0.113)	
(6) Season						
Late hyperphagia	−0.003 (−0.179 to 0.172)				***0.450 (0.021–0.878)***	
Hypophagia	−0.073 (−0.224 to 0.078)				0.286 (−0.081 to 0.653)	
Early hyperphagia	0				0	
(7) Sex × age interaction?	No	No	No	No	No	No
(8) Sex × season interaction?	No	No	No	No	Yes	No

Only coefficients from the top models [Akaike’s information criteria corrected for small sample sizes (ΔAIC_c_) = 0.00] are shown. Those in bold, italicized typeface have credible intervals that do not include zero.

**Table 4:** Standardized coefficients and credible intervals of model variables describing apoptosis and cell cycle protein expression in skin biopsy samples collected from 119 grizzly bears over 139 captures in western Alberta, Canada, from 2004 to 2012

Predictor variables	Apoptosis and cell cycle stress proteins (standardized coefficient and credible interval)							
	AIF	Annexin II	Annexin IV	Caspase 1	Caspase 2	Caspase 6	E-Cadherin	GAPDH
(1) Intercept	***−0.418 (−0.826 to −0.010)***	***−0.577 (−0.924 to 0.230)***	***−0.541 (−0.806 to −0.276)***	***−0.465 (−0.895 to −0.034)***	−0.386 (−0.777 to 0.005)	−0.102 (−3.022 to 2.818)	***−0.480 (−0.905 to −0.056)***	−0.002 (−0.413 to 0.409)
(2) Year								
2012	0.449 (−0.100 to 0.997)	***0.531 (0.093–0.969)***	***0.541 (0.230–0.852)***	0.474 (−0.094 to 1.042)	0.458 (−0.075 to 0.991)	0.633 (−3.492 to 4.758)	0.454 (−0.116 to 1.025)	***−0.605 (−1.150 to 0.059)***
2011	0.443 (−0.116 to 1.002)	***0.536 (0.080–0.991)***	***0.522 (0.188–0.855)***	0.455 (−0.127 to 1.038)	0.486 (−0.057 to 1.028)	0.511 (−3.616 to 4.638)	0.450 (−0.132 to 1.032)	***−0.879 (−1.442 to −0.315)***
2010	0.525 (−0.125 to 1.176)	***0.589 (0.038–1.139)***	***0.589 (0.125–1.053)***	0.456 (−0.240 to 1.152)	0.619 (−0.047 to 1.284)	0.272 (−3.887 to 4.430)	0.530 (−0.171 to 1.230)	***−1.268 (−1.977 to 0.559)***
2008	0.025 (−0.127 to 0.177)	0.052 (−0.120 to 0.223)	0.034 (−0.133 to 0.200)	0.042 (−0.139 to 0.224)	0.073 (−0.060 to 0.205)	−0.094 (−0.257 to 0.070)	0.005 (−0.160 to 0.169)	−0.060 (−0.241 to 0.122)
2007	0.158 (−0.021 to 0.337)	0.100 (−0.100 to 0.300)	0.115 (−0.081 to 0.310)	0.156 (−0.058 to 0.370)	0.100 (−0.046 to 0.246)	0.003 (−0.175 to 0.181)	0.138 (−0.058 to 0.333)	***0.313 (0.114–0.513)***
2006	***0.227 (0.053–0.402)***	***0.198 (0.002–0.394)***	0.178 (−0.013 to 0.369)	0.203 (−0.005 to 0.411)	***0.207 (0.066–0.349)***	0.099 (−0.075 to 0.273)	***0.209 (0.020–0.398)***	***0.240 (0.047–0.434)***
2005	***0.293 (0.127–0.460)***	***0.287 (0.100–0.474)***	***0.270 (0.088–0.452)***	***0.275 (0.075–0.474)***	***0.252 (0.120–0.384)***	0.134 (−0.028 to 0.296)	***0.276 (0.095–0.458)***	***0.325 (0.144–0.505)***
2004	0	0	0	0	0	0	0	0
(3) Bear management area								
Grande Cache	0.007 (−0.166 to 0.179)	0.072 (−0.133 to 0.276)	0.075 (−0.120 to 0.270)	0.040 (−0.169 to 0.248)			0.042 (−0.142 to 0.226)	
Swan Hills	0.097 (−0.136 to 0.331)	0.152 (−0.126 to 0.430)	0.166 (−0.099 to 0.431)	0.143 (−0.140 to 0.425)			0.127 (−0.122 to 0.375)	
Yellowhead	0.093 (−0.101 to 0.288)	0.164 (−0.069 to 0.396)	0.156 (−0.066 to 0.377)	0.138 (−0.098 to 0.373)			0.122 (−0.084 to 0.329)	
Clearwater	0.094 (−0.112 to 0.299)	0.095 (−0.151 to 0.340)	0.074 (−0.160 to 0.308)	0.100 (−0.149 to 0.349)			0.082 (−0.136 to 0.301)	
Livingstone	−0.099 (−0.306 to 0.107)	−0.124 (−0.370 to 0.123)	−0.104 (−0.339 to 0.130)	−0.107 (−0.357 to 0.143)			−0.114 (−0.335 to 0.106)	
Castle	0	0	0	0			0	
(4) Sex								
Male								
Female with young								
Solitary female								
(5) Age								
(6) Season								
Late hyperphagia					0.031 (−0.116 to 0.178)	0.014 (−0.166 to 0.195)		0.069 (−0.133 to 0.271)
Hypophagia					−0.015 (−0.142 to 0.113)	−0.056 (−0.214 to 0.102)		−0.132 (−0.304 to 0.039)
Early hyperphagia					0	0		0
(7) Sex × age interaction?	No	No	No	No	No	No	No	No
(8) Sex × season interaction?	No	No	No	No	No	No	No	No

Only coefficients from the top models (ΔAICc = 0.00) are shown. Those in bold, italicized typeface have credible intervals that do not include zero.

**Table 5:** Model-averaged standardized coefficients and credible intervals of model variables describing cellular stress and proteotoxicity protein expression in skin biopsy samples collected from 119 grizzly bears over 139 captures in western Alberta, Canada, from 2004–2012

Predictor variables	Cellular stress and proteotoxicity proteins (standardized coefficient and credible interval)								
	Cytokeratin	GRP78/BiP	HSP27	HSP40	HSP60	HSP70	HSP70 inducible	HSP90	HSP110
(1) Intercept	0.004 (−0.176 to 0.183)	0.351 (−0.139 to 0.841)	0.353 (−0.284 to 0.989)	***−0.399 (−0.741 to −0.057)***	−0.261 (−0.632 to 0.110)	−0.318 (−1.743 to 1.107)	−0.373 (−0.894 to 0.149)	0.390 (−0.143 to 0.924)	−0.040 (−0.218 to 0.139)
(2) Year									
2012	***0.719 (0.530–0.908)***	−0.279 (−0.926 to ’0.369)	−0.442 (−1.325 to 0.441)	0.326 (−0.128 to 0.781)	***0.544 (0.047–1.041)***	0.550 (−1.457 to 2.558)	0.379 (−0.338 to 1.097)	0.274 (−0.457 to 1.004)	***0.192 (0.018–0.367)***
2011	***0.562 (0.339–0.784)***	−0.486 (−1.149 to ’0.178)	−0.763 (−1.654 to 0.128)	0.199 (−0.270 to 0.669)	***0.588 (0.078–1.099)***	0.462 (−1.549 to 2.473)	0.131 (−0.595 to 0.858)	0.220 (−0.522 to 0.962)	0.026 (−0.177 to 0.229)
2010	***0.562 (0.035–1.088)***	***−0.915 (−1.694 to −0.136)***	***−1.078 (−2.095 to −0.060)***	0.208 (−0.382 to 0.798)	***0.880 (0.198–1.562)***	0.334 (−1.729 to 2.396)	0.209 (−0.671 to 1.089)	0.218 (−0.699 to 1.135)	−0.346 (−0.695 to 0.002)
2008	−0.011 (−0.167 to 0.145)	***−0.212 (−0.413 to ’−0.011)***	***−0.183 (−0.341 to −0.024)***	−0.079 (−0.229 to 0.071)	−0.034 (−0.186 to 0.119)	−0.094 (−0.258 to 0.070)	0.050 (−0.110 to 0.209)	***−0.373 (−0.546 to −0.199)***	−0.074 (−0.220 to 0.071)
2007	−0.018 (−0.189 to 0.153)	***0.270 (0.034–0.506)***	0.043 (−0.130 to 0.215)	0.008 (−0.157 to 0.173)	−0.066 (−0.233 to 0.102)	0.082 (−0.114 to 0.279)	0.077 (−0.096 to 0.251)	−0.164 (−0.353 to 0.024)	***0.232 (0.062–0.403)***
2006	0.130 (−0.036 to 0.297)	0.090 (−0.140 to 0.321)	0.066 (−0.103 to 0.235)	0.126 (−0.034 to 0.286)	***0.191 (0.028–0.353)***	0.157 (−0.032 to 0.346)	0.164 (−0.005 to 0.334)	−0.110 (−0.295 to 0.074)	0.160 (−0.007 to 0.326)
2005	0.081 (−0.073 to 0.236)	0.180 (−0.039 to 0.400)	0.112 (−0.045 to 0.269)	***0.167 (0.0183–0.317)***	0.036 (−0.115 to 0.187)	***0.202 (0.019–0.385)***	***0.231 (0.073–0.389)***	***−0.195 (−0.367 to −0.023)***	***0.246 (0.088–0.405)***
2004	0	0	0	0	0	0	0	0	0
(3) Bear management area									
Grande Cache		−0.180 (−0.412 to 0.052)				−0.012 (−0.190 to 0.167)			−0.156 (−0.326 to 0.014)
Swan Hills		−0.084 (−0.399 to 0.230)				0.076 (−0.160 to 0.311)			−0.070 (−0.301 to 0.161)
Yellowhead		−0.035 (−0.297 to 0.228)				0.080 (−0.116 to 0.275)			−0.008 (−0.201 to 0.185)
Clearwater		0.202 (−0.076 to 0.479)				0.153 (−0.055 to 0.361)			0.140 (−0.064 to 0.344)
Livingstone		−0.174 (−0.452 to 0.104)				−0.087 (−0.301 to 0.127)			−0.161 (−0.366 to 0.043)
Castle		0				0			0
(4) Sex									
Male									
Female with young									
Solitary female									
(5) Age									
(6) Season									
Late hyperphagia	−0.006 (−0.178 to 0.167)		−0.103 (−0.278 to 0.072)	0.039 (−0.128 to 0.205)	0.096 (−0.073 to 0.265)		0.007 (−0.169 to 0.183)	−0.064 (−0.255 to 0.128)	
Hypophagia	−0.040 (−0.191 to 0.110)		***−0.217 (−0.370 to −0.064)***	−0.056 (−0.198 to 0.086)	0.032 (−0.115 to 0.178)		−0.115 (−0.269 to 0.039)	−0.060 (−0.227 to 0.108)	
Early hyperhagia	0		0	0	0		0	0	
(7) Sex × age interaction?	No	No	No	No	No	No	No	No	No
(8) Sex × season interaction?	No	No	No	No	No	No	No	No	No

Only coefficients from the top models (ΔAICc = 0.00) are shown. Those in bold, italicized typeface have credible intervals that do not include zero.

**Table 6:** Standardized coefficients and credible intervals of model variables describing oxidative stress and inflammation protein expression in skin biopsy samples collected from 119 grizzly bears over 139 captures in western Alberta, Canada, from 2004 to 2012

Predictor variables	Oxidative stress and inflammation proteins (standardized coefficient and credible interval)							
	CCR5	COX-2	HO-2	eNOS	iNOS	PRDX3	SOD-1	SOD-2
(1) Intercept	***−0.681 (−1.305 to −0.058)***	−0.423 (−1.412 to 0.566)	−0.426 (−0.911 to 0.058)	−0.221 (−0.547 to 0.105)	0.234 (−0.030 to 0.498)	0.132 (−0.179 to 0.443)	−0.425 (−1.284 to 0.433)	−0.337 (−1.093 to 0.419)
(2) Year								
2012	−0.223 (−0.931 to 0.485)	0.465 (−0.925 to 1.854)	0.377 (−0.288 to 1.042)	0.181 (−0.226 to 0.587)	0.080 (−0.250 to 0.410)	−0.108 (−0.524 to 0.307)	0.412 (−0.790 to 1.615)	0.384 (−0.673 to 1.441)
2011	−0.541 (−1.263 to 0.182)	0.445 (−0.948 to 1.839)	0.385 (−0.288 to 1.058)	0.025 (−0.400 to 0.449)	0.008 (−0.340 to 0.355)	−0.252 (−0.679 to 0.176)	0.425 (−0.782 to 1.632)	0.119 (−0.944 to 1.182)
2010	***−0.940 (−1.812 to −0.068)***	0.646 (−0.816 to 2.108)	0.471 (−0.317 to 1.258)	−0.019 (−0.537 to 0.499)	−0.189 (−0.723 to 0.345)	***−0.618 (−1.171 to 0.064)***	0.601 (−0.687 to 1.889)	−0.052 (−1.214 to 1.110)
2008	−0.084 (−0.289 to 0.122)	−0.021 (−0.163 to 0.121)	−0.016 (−0.165 to 0.134)	0.155 (−0.012 to 0.322)	***−0.200 (−0.357 to −0.043)***	***−0.196 (−0.328 to −0.065)***	−0.031 (−0.190 to 0.129)	0.084 (−0.067 to 0.234)
2007	0.055 (−0.186 to 0.296)	0.146 (−0.009 to 0.301)	0.107 (−0.073 to 0.286)	***0.278 (0.084–0.473)***	0.173 (−0.014 to 0.360)	−0.021 (−0.166 to 0.124)	0.152 (−0.039 to 0.343)	0.069 (−0.095 to 0.233)
2006	0.104 (−0.134 to 0.342)	***0.221 (0.069–0.372)***	***0.182 (0.009–0.355)***	***0.223 (0.033–0.414)***	0.066 (−0.115 to 0.246)	−0.030 (−0.170 to 0.111)	***0.203 (0.019–0.387)***	***0.165 (0.005–0.326)***
2005	***0.240 (0.014–0.466)***	***0.246 (0.105–0.387)***	***0.244 (0.077–0.410)***	***0.308 (0.127–0.490)***	0.127 (−0.047 to 0.300)	0.038 (−0.092 to 0.169)	***0.252 (0.074–0.431)***	***0.209 (0.060–0.359)***
2004	0	0	0	0	0	0	0	0
(3) Bear management area								
Grande Cache	0.198 (−0.046 to 0.442)		−0.002 (−0.167 to 0.162)	0.084 (−0.116 to 0.283)	−0.124 (−0.297 to 0.049)		−0.003 (−0.176 to 0.171)	
Swan Hills	0.225 (−0.113 to 0.562)		0.073 (−0.146 to 0.292)	0.185 (−0.087 to 0.456)	−0.048 (−0.281 to 0.184)		0.082 (−0.147 to 0.312)	
Yellowhead	***0.388 (0.113–0.662)***		0.067 (−0.115 to 0.248)	0.137 (−0.091 to 0.364)	−0.090 (−0.283 to 0.103)		0.095 (−0.096 to 0.285)	
Clearwater	0.278 (−0.009 to 0.565)		0.114 (−0.078 to 0.307)	0.203 (−0.038 to 0.443)	0.182 (−0.023 to 0.386)		0.086 (−0.116 to 0.289)	
Livingstone	−0.010 (−0.290 to 0.269)		−0.109 (−0.305 to 0.088)	−0.089 (−0.330 to 0.152)	−0.103 (−0.310 to 0.104)		−0.107 (−0.315 to 0.101)	
Castle	0		0	0	0		0	
(4) Sex								
Male	***0.530 (0.105–0.955)***							
Female with young	0.306 (−0.098 to 0.710)							
Solitary female	0							
(5) Age	−0.006 (−0.088 to 0.075)							
(6) Season								
Late hyperphagia	0.375 (−0.025 to 0.775)	0.022 (−0.135 to 0.178)				−0.104 (−0.250 to 0.042)		−0.013 (−0.180 to 0.153)
Hypophagia	***0.343 (0.005–0.681)***	−0.024 (−0.16 to 0.113)				***−0.175 (−0.30 to −0.050)***		−0.122 (−0.267 to 0.024)
Early hyperphagia	0	0				0		0
(7) Sex × age interaction?	No	No	No	No	No	No	No	No
(8) Sex × season interaction?	Yes	No	No	No	No	No	No	No

Only coefficients from the top models (ΔAICc = 0.00) are shown. Those in bold, italicized typeface have credible intervals that do not include zero.

Although the geographical location and time model was selected more often than the time-only model as top model (16 vs. 13 times), we could not distinguish clearly between location and time effects because the sampling of locations among years was inconsistent. For example, samples were not obtained from the three southernmost BMAs (Castle, Livingstone and Clearwater BMAs) after 2008. Likewise, the Swan Hills BMA was sampled only in 2005 and 2006.

Irrespective of protein category, the expression of many proteins was greater in 2005 (24 of 31 proteins based on 28 skin samples) and 2006 (13 of 31 proteins based on 21 skin samples) than in 2004 (19 skin samples; Tables 3–6). In 2007 (18 skin samples), protein expression of four proteins was greater than in 2004, but the expression of another 13 proteins was on the borderline (the lower end of the credible interval was slightly <0) of being greater than in 2004. Protein expression was different in 2008 relative to earlier years in that the expression of five proteins, three in the CSP category (Table 5) and two in the OSI category (Table 6), was less than in 2004. From 2010 to 2012, the expression of some proteins was greater (annexin II, annexin IV, cytokeratin, HSP60 and HSP110), whereas the expression of other proteins was less (GAPDH, GRP78/BIP, HSP27, CCR5 and PRDX3) than in 2004 (Tables 4–6). In addition, the credible intervals for protein expression in 2010–2012 were generally two to three times greater than in preceding years.

Among geographical locations, POMC expression was less in samples collected from the Castle and Livingstone BMAs than in samples from other BMAs (Table 3). The expression of CCR5 was also less in samples collected from the Castle BMA than in samples collected from the Yellowhead, and possibly Clearwater and Grande Cache, BMAs (Table 6). The expression of POMC and CCR5 also differed by season, with lowest expression in samples collected during early hyperphagia (15 June to 7 August ). In contrast, HSP27 and PRDX3 expression was greater in samples collected during early hyperphagia than in samples collected at other times of the year (Tables 5 and 6).

Expression of CCR5 in samples collected from male grizzly bears was greater than in samples collected from females (Table 6). However, this sex class difference was most evident during early hyperphagia (i.e. sex × season interaction), but less so during other times of the year. Expression of POMC was also greater in samples collected from males than from females during early hyperphagia, but overall the sex class differences were more subtle for this protein (Table 3).

## Discussion

Protein microarray technologies have made substantial recent progress in areas of human health and drug discovery and have become a dominant method in proteomics research (Chaerkady and Pandey, 2008; Sun *et al*., 2013). However, applications to wildlife conservation are currently lacking. To our knowledge, this study is the first to describe a protein microarray developed specifically for a wildlife species. The need for such an approach arises from the realization that physiological measures of compromised health in individuals, such as measures of stress, can provide early, sensitive warnings that may forecast subsequent impairment of wildlife population dynamics (Clark *et al*., 2001; Wikelski and Cooke, 2006). Given that anthropogenic landscape change is a major threat to wildlife populations worldwide (Lindenmayer and Fischer, 2006), there is a call for the development of novel measures of individual animal physiology and pathology that use modern approaches applied successfully in areas of human biomedical research. The advantages of protein microarrays compared with traditional protein separation and identification techniques, such as enzyme-linked immunosorbent assays, western blotting and two-dimensional gel electrophoresis, include the ability to multiplex protein detection on a single platform, high throughput and reduced sample consumption (Kingsmore, 2006; Sun *et al*., 2013).

The initial development of the grizzly bear protein microarray involved extensive laboratory work to identify a suite of antibodies that cross-reacted with stress-associated proteins in grizzly bear skin. We first attempted to use three commercially available protein microarrays for this purpose, which were designed for basic research in laboratory rodents and for diagnostic purposes in humans (Kopf *et al*., 2005). However, very few antibodies on the commercial arrays cross-reacted with grizzly bear proteins. Further testing of potential antibodies from the commercial arrays using western blotting identified only one antibody (HSP27) that cross-reacted with grizzly bear skin protein. This reinforced the need to test individual commercial antibodies for their ability to recognize grizzly bear proteins of interest. In the end, 31 antibodies of stress-associated proteins were identified and used to create the protein microarray in the present study. The proteins included a variety of hormones, receptors, enzymes and cell signalling molecules that were classified into four broad functional categories associated with physiological stress (HPA axis, ACC, CSP and OSI; Table 1), which are often up-regulated in response to acute and chronic stress.

Inconsistencies in antibody printing and protein capture have been recognized as important factors that limit the use of protein microarray technologies (Kingsmore, 2006; Romanov *et al*., 2014). The microarray described herein displayed excellent precision, with intra-array variation below 10% and inter-array variation below 15% for the majority of proteins. The biopsy devices used in the present study provided between 50 and 100 mg of skin from a single biopsy, which consistently yielded 80 µg of protein to be used in triplicate for each determination. Lesser quantities of protein (10 and 20 µg) produced similar protein expression values, suggesting that smaller skin specimens might also be effective. Experiments with various dilutions of antibody indicated that a 1:1 ratio of antibody to print buffer was optimal. Greater dilutions caused inconsistencies in spot uniformity when scanning, which is a recognized issue with protein microarrays (Romanov *et al*., 2014).

Holding skin samples at ambient (room) temperature for more than 12 h resulted in less protein expression for HPA axis, ACC and OSI proteins, probably as a result of protein degradation. In contrast, expression of CSP proteins was observed to increase with time at room temperature. Given that the majority (six of eight) of proteins in the CSP category belong to heat shock protein (HSP) families (Feder and Hofmann, 1999), this observation may be the result of increases in expression of certain HSPs over the time period at room temperature. Overall, these results suggest that samples collected in the field should be immediately frozen (on dry ice or in liquid nitrogen) if possible, or at least stored on wet ice until freezing on the day of collection. Longer term storage of skin should be in liquid nitrogen or in an ultracold (−80°C) freezer to limit alterations in protein expression.

In many field studies of free-ranging wildlife that use molecular biological approaches involving RNA isolation, small tissue samples (e.g. ear plugs) are commonly stored in a preservative, such as RNA*later*^®^, that stabilizes RNA quality and quantity by inhibiting RNase enzymes. The majority of proteins displayed no significant differences in measured protein expression between skin samples stored in RNA*later*^®^ for 24 h compared with unpreserved samples. This suggests that skin stored in such preservatives can potentially be used to determine protein expression in addition to RNA-based approaches.

Although CSP protein expression was marginally greater in hindlimb compared with other body regions sampled, overall there was consistent measured protein expression among the four body regions sampled in the present study, suggesting that remotely delivered biopsy sampling approaches might be useful in future research (Karesh *et al*., 1987; Pagano *et al*., 2014). Nevertheless, it is recommended that, if possible, skin biopsies be taken from the same location on the body in captured animals to limit any potential variation in protein expression. Importantly, ear plugs provided reliable data on protein expression in comparison to other body regions, suggesting that this commonly collected tissue gathered when ear tags are applied to bears can be used for future research involving the protein microarray.

A GLMM approach was used to identify potential coarse-level effects of bear biology parameters (sex, presence or absence of dependent offspring and age), sampling location (BMA) and season (hypophagia, early hyperphagia or late hyperphagia) on the mean expression of each stress-associated protein. This was intended as a preliminary field validation of the protein microarray, because our ongoing statistical analyses are investigating associations between skin stress protein expression and more specific, fine-level environmental and biological data, including other stress markers (e.g. hair cortisol concentration, serum heat shock proteins and serum corticosteroid-binding globulin), habitat variables (e.g. home range, movement patterns, remote sensing of natural and anthropogenic landscape features and vegetation phenology) and life-history variables. All proteins within each functional category demonstrated differences in expression with respect to year, whereas fewer proteins differed in expression among geographical locations, seasons or sex classes. However, at this coarse level of analysis, we were simply trying to determine whether we could detect patterns to suggest that specific environmental or biological factors might be responsible for observed differences in protein expression. Discussion of the potential physiological basis for these differences is beyond the scope of this ‘proof-’of-concept’ report. Our longer term goal is to apply the protein microarray as a conservation physiology tool that can detect, evaluate and monitor physiological stress in grizzly bears and other species at risk, which will most certainly involve identifying patterns of differential protein expression that can be linked to specific environmental stressors and connecting the underlying physiological processes. Given that the protein microarray can simultaneously determine expression levels of multiple (>30) stress-associated proteins from an individual sample, it has clear advantages over single stress markers (e.g. hair cortisol, serum heat shock proteins or serum corticosteroid binding globulin) in terms of the range of physiological processes that are assessed in the combination of proteins.

Collection of skin from free-ranging wildlife, such as grizzly bears, is relevant from a physiological perspective. As the largest organ in the body of mammals, skin responds to multiple endogenous and exogenous stressors and is thus well suited as a tissue for monitoring stress in wildlife. Importantly, human skin has been reported to possess an independent, functionally equivalent peripheral HPA axis that locally synthesizes and responds to classical trophic factors (e.g. CRH and ACTH), peptide hormones and their precursors (e.g. prolactin and POMC), expresses key steroidogenic enzymes and is subject to negative feedback regulation (Slominski *et al*., 2000; Ito *et al*., 2005; Arck *et al*., 2006). In addition, skin is involved in complex interactions with nervous, endocrine and immune systems (Slominski and Wortsman, 2000). In the present study, expression of a range of proteins associated with the HPA axis and related cellular responses supports the use of skin in studies involving free-ranging wildlife. Further research is needed to establish the functional aspects of stress-associated protein expression in skin from grizzly bears and other wildlife species.

Preliminary work with other wildlife species suggests that the protein microarray described here may be applicable to animals other than grizzly bears. This is not surprising, because we tested a large number of commercial antibodies produced in rodents in order to identify 31 that reliably cross-reacted with grizzly bear skin proteins. To date, we have tested skin protein samples from polar bears (*Ursus maritimus*), bottlenose dolphins (*Tursiops truncatus*), ringed seals (*Pusa hispida*) and moose (*Alces alces*) for their cross-reactivity with antibodies used for the microarray, both by western blotting and on the microarray. In polar bears and bottlenose dolphins, all 31 antibodies cross-reacted specifically and produced protein expression signals on the microarray, whereas 25 of 31 cross-reacted with moose and 24 of 31 cross-reacted with ringed seal. In recent work with bottlenose dolphins, we developed a technique to concentrate and label proteins isolated from white blood cells obtained from the buffy coat layer after blood sampling and centrifugation. All 31 antibodies cross-reacted and produced reliable protein expression signals in dolphin white blood cells using the microarray, indicating that this matrix can also be used when such samples are available. Although a limited number of species have been evaluated to date, these results suggest that the protein microarray may be broadly applicable to many other wildlife species belonging to diverse taxa.

In conclusion, the novel antibody-based microarray developed in this study provides a sensitive and reliable expression profile for a suite of proteins that are involved in a variety of physiological and pathological responses to environmental stressors. In comparison to common approaches that measure single biomarkers of stress, this technique has the primary advantage of, from a single skin biopsy sample, simultaneously determining differences in protein expression arising from multiple pathways associated with cellular stress responses. This may be a useful tool to complement traditional wildlife biology investigations by identifying specific physiological processes being affected by environmental stressors. In addition, the technique shows promise in conservation physiology research involving other species at risk. Our ongoing work is focused on expanding the number of antibodies on the microarray to include other stress-associated proteins, such as acute phase proteins, and proteins associated with energy homeostasis and reproductive physiology. In addition, more comprehensive, finer scale statistical modelling is being performed with a larger number of ancillary predictor variables.

## Funding

This work was supported by the Natural Sciences and Engineering Research Council of Canada (NSERC) Collaborative Research and Development Grant Program (grant number CRDPJ 328937-05); partners of the Foothills Research Institute Grizzly Bear Program; Alberta Innovation and Science; the Western College of Veterinary Medicine (WCVM) Research Trust Fund; and the WCVM Wildlife Health Fund. R.I.C. received an NSERC Postgraduate Scholarship and a scholarship from the Toxicology Graduate Program, University of Saskatchewan.
